# The role of nurses in implementation of public policy on adolescent health in Colombia, Ecuador, and Peru

**DOI:** 10.1186/s12961-024-01134-6

**Published:** 2024-07-04

**Authors:** Silvia Helena De Bortoli Cassiani, Bruna Moreno Dias, Jairo Rivera, Andre Noel Roth Deubel, Taycia Ramírez Pérez, Dinora Rebolledo Malpica, Sonja Caffe

**Affiliations:** 1 Department of Health Systems and Services, Pan American Health Organization, Washington, DC United States of America; 2 Simon Bolivar Andean University, Quito, Ecuador; 3 National University of Colombia, Bogotá, DC Colombia; 4 University of Guayaquil, Guayaquil, Ecuador

**Keywords:** Health policy, Adolescent, Adolescent health, Adolescent development, Nursing, Comprehensive health care, Adolescent health services

## Abstract

**Background:**

In Latin America, interventions aimed at adolescents’ health suffer from a shortfall of investment and lack of sustainability. Nurses, as an integral part of health services and systems, can lead the implementation and development of public health policies to improve adolescent health.

**Objective:**

To identify and analyze the role of nurses in the development and implementation of public policies and in the provision of health care to adolescents in Colombia, Ecuador, and Peru.

**Methods:**

The research was carried out in three phases: a documentary analysis, an online survey, and semi-structured focus groups. A total of 48 documents were analyzed, 288 nurses participated in the survey, and 29 nurses participated in the focus groups.

**Results:**

State policies aim to guarantee rights, with special protection for children and adolescents. It is an incremental process, with greater involvement of civil society and governments. Participants reported a lack of synergy between law and practice, as well as differences in regulatory compliance in rural areas and in populations of different ethnicities and cultures. Their perception was that the protection of adolescents is not specifically enshrined in the legal bases and regulatory structures of the countries, meaning that there are both protective factors and tensions in the regulatory framework. While nurses are highly committed to different actions aimed at adolescents, their participation in policy development and implementation is low, with barriers related to a lack of specialized training and working conditions.

**Conclusions:**

Given nurses’ involvement in different actions aimed at adolescents, they could play a fundamental role in the development of policies for adolescents and ensure their effective implementation. Policymakers should consider revising the budget to make compliance viable, incorporating and using monitoring indicators, and increasing the involvement of educational institutions and the community.

## Background

Adolescence is an important stage of human development. Adolescents, who are neither children nor young adults, face factors, such as physical growth, hormonal changes, sexual development, new emotions, expansion of cognitive skills, and moral and relationship development [1]. Adolescent care involves communication approaches and care skills specific to this stage of growth and development. Health professionals and services must therefore develop competencies and skills to facilitate timely and effective care for this population group, so that they do not become a barrier to progress in universal access to health and universal health coverage [2]. In other words, healthcare services for adolescents must take into account the specificities of adolescents, be accessible, appropriate and timely, determined according to their needs, and without discrimination of any kind, especially for groups in conditions of vulnerability [3–5].

The Pan American Health Organization (PAHO), in its Plan of Action for Women’s, Children’s and Adolescents’ Health 2018–2030, highlights that, despite the advancements observed in recent years, progress in the Region has been unequal and does not benefit some national population subgroups [6]. Adolescent physical, cognitive, psychological, and behavioral development has been limited in population groups in situations of vulnerability who suffer most heavily from preventable mortality and morbidity, preventable diseases, and risk factors [6].

There is evidence that services for adolescents are highly fragmented, poorly coordinated, and uneven in quality [4]. There are global standards for quality required in the provision of services to meet the needs of adolescents, which include: adolescents’ health literacy, community support, appropriate package of services, providers’ competencies, facility characteristics, equity and nondiscrimination, data and quality improvement, and adolescents’ participation [4]. In this sense, policymakers must advocate for public policies aimed at this group that enable health services to be responsive to these standards to promote, protect, and improve the health and well-being of adolescents [4].

In Latin America, interventions aimed at adolescents suffer from a shortfall of public financial and political investment and a lack of sustainability of actions to ensure that systems and services respond to their needs [7]. Having health services that act in an intersectoral manner and create a welcoming space for adolescents is a challenging goal for several reasons, such as having human resources for health (HRH) available in adequate numbers, accessible and adequately distributed health services, and appropriately trained personnel to perform adolescent care [8].

Nurses represent 56% of all health professions, comprising the largest occupational group in the health workforce [9]. They work in health systems, performing leadership, educational, practice, research, and community action roles [6]. Nurses, as an integral part of health services and systems, can lead the implementation and development of public health policies allowing countries to guarantee universal health and achieve the Sustainable Development Goals (SDG), conceptualized as a renewed commitment on the part of all United Nations (UN) Member States to pursue economic, social, and environmental development in a sustainable and equitable manner [9, 10].

Across various levels of action and decision-making, nurses can advocate for adolescent care, especially for populations in situations of vulnerability. Therefore, it is important to evaluate the role of nurses in adolescent health care policies, primarily from their perspectives [9].

To analyze the contributions of nurses regarding use of their knowledge and skills in the implementation public health policies and advocacy for adolescent health care, this study aims to identify and analyze the role of nurses in the development and implementation of public policies and in the provision of health care to adolescents in Colombia, Ecuador, and Peru.

## Methods

### Methodological design and study setting

A multi-method study, carried out in three phases, with qualitative and quantitative analyses, as described below. Three Latin American countries were selected—Colombia, Ecuador, and Peru—with the aim of drawing parallels with other countries in the region, with a similar profile in terms of health systems and populations in situations of vulnerability [11].

#### Phase 1. Analysis of policy documents

The first phase consisted of a documentary analysis to identify and analyze adolescent-related government policies. Current legal documents produced by the state—such as laws, policies, regulations, and decrees—were considered eligible, with no restrictions on year or on the sector responsible for publication (e.g., ministry of health, ministry of women and vulnerable populations, national assembly, etc.).

An instrument developed by policy analysis experts was used, with questions structured according to the elements of the Walt and Gilson triangle (context, content, actors, and process) [12], which has been widely used in public health policy analysis [13–16].

In Walt and Gilson’s triangle, policy analysis takes place in: (a) the historical, political, economic, social, and cultural context; (b) the technical content, objectives, and strategies; (c) the political process and organizational capacity; and (d) at the center are the actors, such as the authorities, the public and private sectors, civil society, and the groups affected and benefiting from the policies, with their values and interests [12, 13].

Data collection was carried out by a duly trained working group, under the supervision and support of two policy analysis experts. A total of 48 documents were identified and analyzed: 18 from Colombia, 15 from Ecuador, and 15 from Peru.

#### Phase 2. Survey

The survey was conducted in the quantitative phase of the study, using a cross-sectional observational design. A questionnaire was applied containing a section on subject demographics, with nine questions and 31 items on a 5-point Likert scale (strongly disagree, disagree, neither agree nor disagree, agree, strongly agree) relating to working conditions, behavioral patterns, and conflicts (real versus ideal service) for understanding the policy analysis and the role of nurses in adolescent care. Data collection was carried out using Survey Monkey, between 11 May and 8 June 2022.

Registered nurses working in different health services and institutions providing adolescent care, such as public health services, community and domiciliary care, general and specialized clinics, educational institutions, and hospitals, were invited to participate. Recruitment was carried out by widely publicizing the invitation in all health services that provide care to adolescents, with the support of the national nurses’ association and national universities. Participants were selected in a convenience sample, with an inclusion criterion of working in services that provide adolescent care. In total, 144 participants who did not complete the questionnaire were excluded. The final sample comprised 288 nurses: 99 from Colombia, 97 from Ecuador, and 92 from Peru. All participants agreed to take part in the study by registering their consent on the online form. Participation was voluntary, with no cost, obligation, or payment of any kind.

#### Phase 3. Focus group

In the qualitative phase of the study, semi-structured focus groups were conducted, with a convenience sample, taking into account the representation of relevant institutions and geographical regions. Groups were conducted in the three countries until theoretical saturation [17].

Participants were identified and recruited through the support of different national stakeholders to compose a group with representatives from different sectors and institutions, such as leaders of child and adolescent health programs, representatives from the health department, the national nurses’ association, universities, adolescent health centers, general and specialized clinics, hospitals, and school nurses. Nurses with at least 5 years of experience working with adolescents were included in order to have a sample of participants with greater experience and understanding of the role of nurses and the organization and functioning of the health system. A total of 29 nurses participated: 13 from Colombia, 8 from Ecuador, and 8 from Peru. All participants agreed to take part in the study by registering their consent on the online form. Participation was voluntary, with no cost, obligation, or payment of any kind.

To carry out the focus group, guiding questions on working conditions, behavioral patterns, and conflicts (real versus ideal service) were used [18], addressing topics, such as the role, involvement, and responsibility of nurses in actions aimed at adolescents, the challenges faced, experiences in applying regulations and policies, and working conditions and resources for adolescent care.

The meetings were held virtually, using the Zoom Meetings tool, in the period between 30 June and 8 July 2022. Four groups were held, two in Colombia, one in Ecuador, and one in Peru. All groups were led by two moderators, nurse researchers with extensive experience in conducting qualitative studies, with the participation and collaboration of researchers from the respective country. The meetings, with an average duration of 90 min, were audio and video recorded. The focus group records were transcribed, and the data were categorized and analyzed together with the researchers’ notes, using content analysis on the basis of the theoretical framework of Walt and Gilson (context, content, actors, and process) [12],

## Results

### Analysis of policy documents

Public policies for children and adolescents in Colombia, Ecuador, and Peru are based on regulations emanating from each country’s constitution, enacting the rights, guarantees, and special protection for this group. In turn, the Convention on the Rights of the Child has been ratified in these countries, and laws and programs have been created to promote the physical, cognitive, psychological, and behavioral development of children and adolescents. This process is spearheaded by public policies from the state, with greater inclusion of civil society, incorporating territorial elements. Table 1 presents the synthesis of context, content, processes, and actors of the documents evaluated per country.

**Table 1:** Synthesis of elements from the analysis of context, content, process, and actors related to public policies on children and adolescents

	Colombia	Ecuador	Peru
Context	Colombia adopted a new Political Constitution in 1991. In the same year, it ratified the 1989 UN Convention on the Rights of the Child. For the health sector, the change resulted in the privatization reform of Law 100 of 1993. For children and adolescents, Law 1098, or the Code of Children and Adolescents, was approved in 2006 (amended in 2018) and the National Policy for Children and Adolescents (2018–2030; PNIA) was established in 2018 to integrate sectoral advancements. Children and adolescents comprised 22% of the country’s population in 2017, half of them in a situation of multidimensional poverty	In 1990, Ecuador ratified the Convention on the Rights of the Child; in 2006, it developed an Organic Health Law. A new constitution was adopted in 2008, with social guarantees and rights for the entire population, including children and adolescents as a priority group. In 2021, the Opportunity Creation Plan was established. In 2019, children and adolescents totaled 6 million people, around 35% of the population, 42% of which were in multidimensional poverty	Peru ratified the Convention on the Rights of the Child in 1989. It adopted a new Constitution in 1993. In 1996, it established the Ministry of Women, with a Directorate-General of Children and Adolescents. It established the General Health Law in 1997. In 2000, it issued the Code for Children and Adolescents, which provides comprehensive protection for this group. In 2021, the National Multisectoral Policy for Children and Adolescents to 2030 was created, in which children and adolescents represent around 8 million people, around 30% of its population
Contents	The 1991 Constitution establishes that children and adolescents are “entitled to protection and integral development” (Art. 45). The 2006 Code on Children and Adolescents (amended in 2018) seeks to guarantee their rights and establishes substantive and procedural regulations for the comprehensive protection of this population. The National Policy for Children and Adolescents—PNIA—(2018–2030) defines strategic actions that are related to national goals in compliance with Sustainable Development Goals, the recommendations of the International Convention on the Rights of the Child, and the major commitments in the 10-year plans on health and education. In general, the policies and regulations set ambitious objectives, regulate health care, and delegate their implementation to territorial entities (decentralization) and the private sector (outsourcing)	Art. 44 of the 2008 Constitution states that “the State, society and the family shall promote as a priority the integral development of children and adolescents and shall guarantee the full exercise of their rights; the principle of the higher interest of children shall be upheld and their rights shall prevail over those of other persons.” The 2003 Code on Children and Adolescents establishes the priority of caring for children and adolescents, where the state, society, and the family must act to guarantee their rights. The 2021 Opportunity Creation Plan is the current national development plan, which proposes to protect families, guarantee their rights and services, eradicate poverty, and promote social inclusion. Within this framework, the plan promotes deconcentration of public services in the territory and decentralization to local governments	Article 4 of the 1993 Constitution states that “the community and the State extend special protection to children, adolescents, mothers, and the elderly in situation of abandonment.” The 1997 General Health Law establishes that it is the responsibility of the State to monitor, protect, and attend to the health problems of the various age groups. The 2000 Code mentions that children and adolescents are subject to rights, freedoms, and specific protection, considering the principle of best interest. The 2021 National Multisectoral Policy for Children and Adolescents to 2030 establishes guidelines to improve the living conditions of children and adolescents. At the local level, there is a deconcentration towards local directorates and local government competencies
Process	The adoption of the Code on Children and Adolescents generated an incremental process of new public policies. While the PNIA was built on an important participatory process, its implementation is based on a top-down process	Public policies for children and adolescents involve greater participation of society, although a vision determined by the governing entity still prevails. There are challenges in combining public, private, and civil society efforts; one example is the teenage pregnancy prevention policy, where there have been advances and setbacks	Public policies in Peru show an evolution towards greater inclusion of society. The National Multisectoral Policy for Children and Adolescents to 2030 has the advantage of incorporating the new challenges posed by the COVID-19 pandemic, which include updated goals to improve the situation of children and adolescents
Actors	According to the Code, the President is responsible for national policy design, implementation, and evaluation through the National Family Welfare System (SNBF). The SNBF comprises a set of agents and coordination efforts to ensure the comprehensive protection of this population at the national, departmental, district, and municipal levels. The Colombian Family Welfare Institute (ICBF), as an entity attached to the Administrative Department for Social Prosperity, is the governing body of the SNBF. At the territorial level, Social Policy Councils are responsible for planning, coordinating, and monitoring the implementation of the PNIA	The main actors include the President, Ministry of Public Health, Ministry of Education, Ministry of Economic and Social Inclusion, Ministry of Finance, National Council for Equality, decentralized autonomous governments, Ministry of Labor, communities, families, and adolescents. The executive branch oversees the management of public health policies, under the leadership of the Ministry of Health. The National Councils for Equality have been created to guarantee that rights are exercised in the territory	The main actors include the President, the National System of Comprehensive Care for Children and Adolescents, the Ministry for the Promotion of Women and Human Development, the Office of the Public Prosecutor, the Ministry of Health, the Ministry of Justice, the Ministry of Labor, regional governments, local governments, communities, families, and adolescents. The governing body for children and adolescents is the Ministry for the Promotion of Women and Human Development, with various ministries having competencies at the local level

**Fig. 1:**
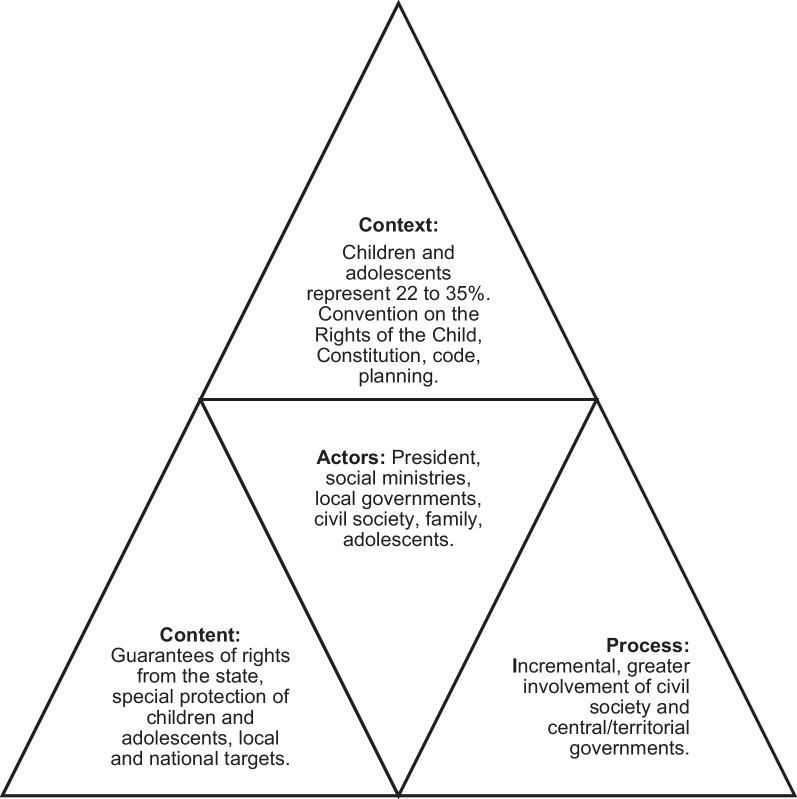
Context, content, process, and actors related to public policies on children and adolescents in Colombia, Ecuador, and Peru

Although each country has its own unique context, content, process, and actors, in general, all demonstrate a firm intention to protect children and adolescents. Figure 1 summarizes this.

### Analysis of nurses’ opinions

The majority of participants were women (89.6%), between 45 and 54 years of age (27.1%). In terms of professional profile, 35.1% had a master’s degree (highest level of education) and had been working in nursing for more than 20 years (39.9%). A total of 81.9% worked full-time; 40.3% worked in an inpatient care service, 29.5% in public health, and 23.6% in an educational institution.

In the nurses’ opinion, the services provided to adolescents follow current regulations and policies (62.8%). Between 44.8% and 49.3% of nurses said that the policies and regulations consider the political, social, cultural, and population contexts in situations of vulnerability, with economic context being the least considered (38.2%; Table 2).

**Table 2:** Nurses’ perception of practices related to adolescent care (*n* = 288)

	Strongly disagree	Disagree	Neither agree nor disagree	Agree	Strongly agree
The service in which I work provides me with adequate working conditions for adolescent care	17 (5.9)	56 (19.4)	69 (24.0)	113 (39.2)	33 (11.5)
The health service in which I work has an adequate infrastructure for adolescent care	31 (10.8)	81 (28.1)	56 (19.4)	97 (33.7)	23 (8.0)
The service in which I work has the institutional and organizational capacity for adolescent care	22 (7.6)	71 (24.7)	61 (21.2)	103 (35.8)	31 (10.8)
The services provided to adolescents follow current regulations and policies	11 (3.8)	40 (13.9)	56 (19.4)	144 (50.0)	37 (12.8)
My manager and/or administrators provide adequate support to turn current adolescent health policies into action	18 (6.3)	58 (20.1)	69 (24.0)	113 (39.2)	30 (10.4)
Education and training activities related to current adolescent health policies are offered	21 (7.3)	59 (20.5)	61 (21.2)	116 (40.3)	31 (10.8)
Updates to adolescent health policies and regulations are communicated to all health professionals and services	21 (7.3)	75 (26.0)	64 (22.2)	105 (36.5)	23 (8.0)
Adolescent health policies and regulations are frequently updated	18 (6.3)	83 (28.8)	79 (27.4)	90 (31.3)	18 (6.3)
Adolescent health policies and regulations are effective	17 (5.9)	78 (27.1)	90 (31.3)	87 (30.2)	16 (5.6)
The budget allocated to the implementation of adolescent health programs is satisfactory to achieve the proposed objectives	38 (13.2)	111 (38.5)	78 (27.1)	50 (17.4)	11 (3.8)
There is systematic monitoring of indicators related to adolescent health	23 (8.0)	85 (29.5)	81 (28.1)	83 (28.8)	16 (5.6)
Adolescent health policies and regulations consider: 1. Political context	18 (6.3)	62 (21.5)	79 (27.4)	113 (39.2)	16 (5.6)
2. Economic context	21 (7.3)	83 (28.8)	74 (25.7)	91 (31.6)	19 (6.6)
3. Social context	19 (6.6)	66 (22.9)	61 (21.2)	123 (42.7)	19 (6.6)
4. Cultural context	17 (5.9)	66 (22.9)	65 (22.6)	119 (41.3)	21 (7.3)
5. Populations in situations of vulnerability	22 (7.6)	59 (20.5)	78 (27.1)	106 (36.8)	23 (8.0)
The following entities are heavily involved in actions aimed at adolescents in situations of vulnerability: 1. National government	23 (7.9)	87 (30.2)	65 (22.6)	88 (30.6)	25 (8.7)
2. Local government	19 (6.6)	83 (28.8)	70 (24.3)	97 (33.7)	19 (6.6)
3. Healthcare professional associations	19 (6.6)	64 (22.2)	78 (27.1)	108 (37.5)	19 (6.6)
4. Schools/educational institutions	16 (5.6)	51 (17.7)	66 (22.9)	128 (44.4)	27 (9.4)
5. Private sector	23 (8.0)	79 (27.4)	83 (28.8)	86 (29.9)	17 (5.9)
6. International organizations	15 (5.2)	63 (21.9)	95 (33.0)	93 (32.3)	22 (7.6)
7. Nongovernmental organizations	13 (4.5)	66 (22.9)	101 (35.1)	88 (30.6)	20 (6.9)
8. Civil servants/policymakers	22 (7.6)	80 (27.8)	83 (28.8)	81 (28.1)	22 (7.6)
9. Community	13 (4.5)	72 (25)	76 (26.4)	107 (37.2)	20 (6.9)
Nurses are heavily involved in the following actions aimed at adolescents: 1. Defining adolescent problems/needs	13 (4.5)	51 (17.7)	56 (19.4)	128 (44.4)	40 (13.9)
2. Finding solutions	12 (4.2)	53 (18.4)	64 (22.2)	128 (44.4)	31 (10.8)
3. Decision-making	15 (5.2)	57 (19.8)	61 (21.1)	124 (43.1)	31 (10.8)
4. Implementing actions	10 (3.5)	50 (17.4)	62 (21.5)	127 (44.1)	39 (13.5)
5. Evaluation	10 (3.5)	56 (19.4)	67 (23.3)	122 (42.4)	33 (11.5)
Nurses are involved in the development of public health policies aimed at adolescents	24 (8.3)	69 (24.0)	74 (25.7)	93 (32.3)	28 (9.7)

Almost half of the participants responded that the service provides adequate conditions for adolescent care (50.7%), that nurses have managerial and administrative support to implement actions (49.7%), and that training activities related to current policies are offered (51.0%).

There is less consensus on adequate infrastructure for adolescent care, and the updating and effectiveness of policies and regulations, with similar scores for negative perceptions and positive ones. The budget allocated to the implementation of adolescent programs delivery (51.7%) and the systematic monitoring of indicators related to adolescent health (37.5%) were negatively evaluated by the participants.

Among the actors, educational institutions (53.8%), communities (44.1%), health care professional associations (44.1%), and local government (40.3%) were those most cited as being involved in actions aimed at adolescents; the private sector and civil servants/political representatives, on the other hand, were those least cited as being involved (35.8%).

Nurse involvement in policy development was affirmed by 42% of participants, with professionals seen as heavily involved in defining adolescent problems/needs (58.3%), drafting a solution (55.2%), decision-making (53.8%), implementing actions (57.6%), and evaluation (53.8%).

The theoretical structure of regulatory frameworks is clear and well founded, facilitating their applicability. However, subjects mentioned that the protection of adolescents is not specifically codified in the legal bases and regulatory structure of the countries, meaning that there are protective factors and tensions in the health regulatory framework.

“The exclusive protection of adolescents is not clarified, and it is difficult to apply specific policies and regulations for adolescents” PE1.

“The law does not protect pregnant adolescents in the same way as pregnant women of other ages, and they even lose their schooling” PE2.

The regulatory framework is broader from a territorial perspective, addressing the local context; however, subjects mentioned that *“the territorial realities in practices with adolescents do not resemble those carried out in the country’s cities where there are no resources”* PC3.

In that regard, some participants noted that there is variance in the level of actual compliance with established rules in rural areas and in populations with diverse ethnicities and cultural backgrounds; this is related to the lack of resources and greater difficulties in accessing health services that are sensitive and respectful of cultural particularities and have the capacity to communicate and share knowledge with the people of these communities.

Participants mentioned the lack of interconnection between different actors (national government, local government, health care professional associations, schools/educational institutions, private sector, international organizations, nongovernmental organizations, civil servants/policymakers, and community) and levels of the health system, with the breaking of procedure regulations and protocols, leading to a lack of synergy between law and practice.

“The efforts of some institutions and professionals are not coordinated with other actors within the health system and much less with others outside this sector, making it impossible to do intersectoral work, which is essential for health systems to function optimally” PC4.

Nurses working in adolescent care are committed to the provision of differentiated and comprehensive care with an intercultural approach that is consistent with the vulnerabilities of adolescents. This is a facilitating factor of the professional role. Likewise, nurses are empowered to provide adolescent care on the basis of an environment of trust, assertive communication, and ethics, developing an educational role and individualized adolescent care, which is humanized in turn. However, the resources for adolescent care, which include inadequate infrastructure (structural characteristics of health facilities) for adolescent care, constitute barriers to accessibility and care with dignity, privacy, quality, and warmth. Nurses reported challenges managing resources and supplies to guarantee optimal care. They face job insecurity owing to an overload of activities, inadequate physical spaces and equipment, and a lack of supplies, such as medicines and materials suitable for service delivery. Some participants even reported situations in which they had to pay out of pocket for supplies and materials to implement activities aimed at adolescents.

“The nurse–patient ratio is challenging; there is a lack of infrastructure in public institutions and a shortage of supplies for care” PE6.

“We have even had to use personal resources to support adolescent care, so as not to lose continuity in the care processes” PC7.

“In public services, we often do not have the infrastructure and equipment to provide care with privacy” PC8.

When asked about their understanding of nurses’ role in adolescent care, participants provided their perception of care. The nurses highlighted that adolescent care needs are specialized, and does not simply fall under general care. Specifically, they noted that they should receive specialized training on adolescents.

Participants affirmed that caring for adolescents in current times is challenging and demanding owing to the highly complex situations of vulnerability they face, which place them in a position of risk in guaranteeing access to actions and services on the basis of citizenship. However, they feel committed to care and have *“expectations regarding the improvement of academic training, and recognition of the leading role of nursing”* PP10.

In this regard, participants acknowledge the commitment to adolescent care.

“We are aware of the importance of assessing and identifying needs in adolescents. It is part of our responsibility as caregivers, and we are clear about that” PC11.

At the same time, nurses’ well-being—and consequently, their outcomes—are hindered by staff dissatisfaction owing to lack of financial recognition, reported in late payments, and nurses’ perception of inadequate pay for work, as well as a lack of moral and legal support in their professional practice, and very unequal working and hiring conditions, in terms of contractual arrangements and guarantee of labor rights.

“I was working in an institution that didn’t pay me for months, but ironically it did demand that I be up to date with their social security payments” PC13.

“The system needs us to be efficient; it doesn’t matter how we do it, but we need the system to work” PC14.

“The salary is often terrible. Sometimes, you’re looking for work and come across salaries that are truly disheartening” PP15.

## Discussion

Adolescents in Latin America and face multiple dimensions of vulnerability, with concrete threats to their prospects for survival and a healthy life. An adequate response requires a comprehensive approach and the strengthening of intersectoral actions and strategies that help adolescents to thrive and access social services [19].

Nurses are clearly committed to adolescent health and development, and they are seen as responsible for adolescent care. They are involved in a range of actions that include determining problems and needs, finding solutions, decision-making, and implementation, even though they do not feel like the main actors in the care of this group.

Despite the potential contributions of nurses, there is evidence that nurses’ participation in policymaking has not increased in recent years. Institutions and regulatory bodies should prepare and encourage nurses to train and facilitate their participation in policy development, not just policy implementers and advocates [20].

In addition to capacity building, nurses’ greatest participation in the political sphere will be through identifying different actors, both formal and informal, and recognizing the different spaces in which policy takes place (organizations; health systems; local, national, or international level) [21].

As this study highlights, it is essential to recognize other actors, such as the educational sector and communities. The uniting of different actors will strengthen actions benefiting adolescent well-being and health.

Intersectoral actions between education and health facilitate adolescent well-being, the development of social skills, and employability, allowing those who live in situations of vulnerability to have the opportunity to change direction [22].

In this regard, given that schools/educational institutions were considered to be the entities most heavily involved in actions aimed at adolescents, the adoption of school nurses is recommended. School nurses can support adolescents by establishing an effective bond, listening carefully to their feelings and health needs, and intervening early and continuously, in collaboration with the family, school, and health services, on the basis of comprehensive care [23]. From an educational system perspective, school nurses have been shown to contribute to improving health, reducing absenteeism, and improving students’ school performance [24]. For this reason, the World Health Organization (WHO) supports the creation of comprehensive school health services as an important community resource to address the health needs of adolescents and increase their participation in health services [25].

Adolescents themselves are other important actors in this process. Adolescents should be involved in the design, implementation, and evaluation of interventions targeting this group [26].

Implementation structure and policy monitoring are key takeaways from this study. There is a limited budget allocated for the care of this group; in real terms, this means a shortage of supplies and job insecurity for health professionals, including nurses. In addition, there is little systematic monitoring of policy compliance; the theoretical framework is clear and precise, but it is not operational. As well as investing in and guaranteeing adequate resources for adolescent policies, teams and nurses must have organizational capacity, infrastructure support, and be able to access, understand, and use data for policy development and implementation [27].

Finally, it is important to note that the well-being of adolescents in situations of vulnerability will continue to be neglected until the key actors identify and prioritize their needs. This requires adequate investments and resources. Financial and political commitment is essential to support the diverse needs of adolescents [26].

Health professionals play a key role in health systems. Without the necessary interventions in working conditions, financial recognition, and adequate infrastructure, policy implementation may not be as effective as expected.

## Conclusions

Nurses are involved in various aspects of the policy implementation process and can make important contributions to child and adolescent health. They take responsibility for and are committed to adolescent care, however, the lack of specialized training in adolescent care and poor working conditions are possible barriers to effective care.

Access to care and the guarantee of rights is pursued by government policies, aimed at providing special protection for children and adolescents, and achieving national and local goals. It is an incremental process, with greater inclusion of civil society and State involvement. The main actors are government (president, social ministries, local governments) and civil society (family, adolescents).

For more effective programs aimed at adolescents, policymakers should consider revising the budget to make it viable, including management of resources, supplies, infrastructure, and equipment to guarantee adequate care. In addition, there is a need to incorporate monitoring indicators and ensure greater participation from educational institutions and the community.

There is a lack of synergy between regulations and practice, as well as differences in regulatory compliance in rural areas and in different ethnic and cultural groups.

Given nurses’ involvement in different actions aimed at adolescents, they could play a bigger role in developing policies and implementing regulations benefiting adolescents. Increased investment in professional training, guaranteed continuing education as needed, and improved working conditions will surely result in better population health outcomes.

## Data Availability

The datasets generated and/or analyzed during the current study are not publicly available owing to privacy issues but are available from the corresponding author on reasonable request.

## Abbreviations

PAHO: Pan American Health Organization
PC: Participant from Colombia
PE: Participant from Ecuador
PP: Participant from Peru
SDG: Sustainable development goals
WHO: World Health Organization
